# Association between anemia and bronchopulmonary dysplasia in preterm infants

**DOI:** 10.1038/srep22717

**Published:** 2016-03-03

**Authors:** Jun Duan, Xiangyong Kong, Qiuping Li, Shaodong Hua, Sheng Zhang, Xiaoying Zhang, Zhichun Feng

**Affiliations:** 1 Department of Pediatrics, BaYi Children’s Hospital Affiliated to Clinical Medical College in Beijing Military General Hospital of Southern Medical University, Beijing 100700, China

## Abstract

Anemia is commonly seen in preterm infants. It may reduce the capacity of hemoglobin to transport oxygen throughout the body and may result in tissue and organ dysfunction. This study aimed to investigate the effect of anemia on the development of bronchopulmonary dysplasia (BPD) in preterm infants. 243 infants who were admitted to BaYi Children’s Hospital Affiliated to Clinical Medical College in Beijing Military General Hospital with gestational age (GA) less than 32 weeks from February, 2014 to February, 2015 were included in the study. Maternal and infant data were recorded. Multivarariate logistic regression analysis was performed to determine the association between anemia and BPD. Of 243 preterm infants, the incidence of anemia was higher in BPD patients than non-BPD patients (*p* < 0.001). Mean Hct in BPD patients was lower than non-BPD patients at different time points in 1d, 7d, 14d, and 21d. Controlling for other confounding factors, early anemia was associated with an increased risk of BPD. Number of transfusions is also a significant risk factor for BPD (*p* = 0.001). Therefore, prevention and treatment of early anemia is necessary and reducing number of transfusions may reduce the incidence of BPD in preterm infants.

Bronchopulmonary dysplasia (BPD) is the most common chronic lung disease in preterm infants. In the past years, remarkable advances in perinatal care including prenatal steroid therapy, surfactant use and improved ventilator strategy, have led to increased survival rates of preterm infants^1^,^2^,^3^. However, the incidence of BPD has not declined. The pathogenesis of BPD has been studied by animal studies and human studies^4^. So far, a number of potential risk factors have been reported to be associated with the development of BPD, including premature birth, low birth weight (BW), genetic predisposition, prolonged mechanical ventilation , hyperoxia, infections, and patent ductus arteriosus (PDA)^5^,^6^,^7^,^8^,^9^,^10^,^11^,^12^,^13^.

Anemia is commonly seen in preterm infants^14^. The pathogenesis is usually multifactorial; it can result from inadequate erythropoietin response, rapid growth, and repeated blood sampling for laboratory tests^15^,^16^. Symptoms of anemia include lactic acidosis, tachycardia, bradycardia, tachypnea, apnea, poor weight gain, diminished activity, and oxygen requirement^17^,^18^. Nevertheless, the effect of anemia on BPD has not yet been investigated yet.

We performed a prospective study to investigate the association of anemia with the development of BPD in preterm infants. As low Hct represents the hallmark of anemia, we recorded Hct at different time points. We also investigated whether early anemia affected the development of BPD.

## Methods

### Study population

An observational study was conducted to determine the effect of anemia on the development of BPD. The study population included 248 infants with gestational age (GA) <32 weeks admitted to the Neonatal Intensive Care Unit (NICU) of BaYi Children’s Hospital Affiliated to Clinical Medical College in Beijing Military General between February 2014 and February, 2015. Infants with major congenital anomalies or syndromes, those who died before 36 weeks’ postmenstrual age (PMA) were excluded from the study. The study was approved by the ethics committees of Beijing Military General Hospital. The methods were carried out in accordance with the approved guidelines. Informed consent of the parents was obtained before the study.

### Data collection

Every infant was followed during their initial hospitalization until time of discharge. Diseases were diagnosed by experienced doctors according to standard definitions. Maternal and infant data were collected from medical records by trained research staff. Maternal data included pregnancy-induced hypertension, maternal diabetes, maternal age, premature rupture of membranes, use of antibiotics and use of prenatal steroids. Infant data included GA, BW, gender, mode of delivery, time of umbilical cord clamping, Apgar scores at 1, 5, and 10 min, surfactant use, days of mechanical ventilation, pneumonia, PDA, sepsis, necrotizing enterocolitis (NEC), retinopathy of prematurity (ROP), intaventricular hemorrhage (IVH), number of transfusions, and length of hospital stay. Gestational age was defined as the number of completed weeks from the first day of the mother’s last menstrual period to the day of delivery. Pneumonia was diagnosed by clinical symptoms and chest X-ray. PDA was diagnosed by two-dimensional color Doppler examination in the first 2 weeks of life. Sepsis was diagnosed by the presence of positive blood cultures during the hospital stay. NEC was diagnosed according to modified Bell’s criteria^19^. ROP was diagnosed according to the international classification of retinopathy of prematurity^20^. IVH was identified by serial cranial ultrasound.

Blood samples (1.0ml) were collected from the infants at 1d, 7d, 14d, 21d, and 28d of postnatal life by venous blood sampling and sent immediately to the Central Laboratory of the Hospital for measurement of Hct. Anemia was defined as Hct <39%^21^. We categorized early anemia as occurring at ≤14 days of postnatal life and late anemia as occurring >14 days.

The main treatment for anemia in our hospital was transfusion. The indication of transfusion in our hospital was as follows: (1) Hct <20% with low reticulocyte count and symptoms of anemia; (2) Hct <30% in an infant with a fraction of inspired oxygen (FiO_2_) <30% on continuous positive airway pressure (CPAP) and/or intermittent mandatory mechanical ventilation with mean airway pressure (MAP) <8 cm H_2_O; with significant apnea, bradycardia, tachycardia or tachypnea; (3) Hct <35% in an infant with FiO_2_ >30% on CPAP/intermittent mandatory mechanical ventilation with MAP ≥8 cm H_2_O. When required, 15 ml/kg packed red blood cells was given to infants. The target Hct was ≥40%. Recombinant human erythropietin (rHuEPO) was not used in our hospital. All preterm infants were given iron supplementation with a dose of 2 mg/kg/d after 1 month of postnatal life.

BPD was defined for infants born at GA less than 32 weeks and treatment with supplemental oxygen more than 28 days by the National Institute of Child Health and Human Development (NICHD)^22^. BPD was classified as mild, moderate and severe based on the required fraction of inspired oxygen at 36 weeks’ PMA: mild, no supplemental oxygen; moderate, supplemental oxygen ≤30%; and severe, the need for mechanical ventilation and/or oxygen >30%^22^.

### Sample size and statistical analyses

Before the study, we calculated that a sample size of at least 221 infants was required to achieve at 2-sided significance level of 0.05 and power of 80%. Median (range) or Mean ± standard deviation (SD) was presented for continuous variables. Kolmogorov-Smirnov test was used to test normality of the data. Two-sample student’s two-tailed t-test was used to compare normally distributed continuous variables, the Mann-Whitney rank-sum two-tailed test was used to compare non-normally distributed continuous variables and Pearson’s exact chi-square test was performed to compare categorical variables. Risk factors that were significant in the univariate analysis were selected as covariates and BPD as dependent in the multiple logistic regression models to obtain adjusted odds ratio (OR) and 95% confidence interval (CI) by using Enter method. A *p* value of less than 0.05 was considered to indicate significance. Data were analyzed using SPSS 13.0 software (SPSS, Inc., Chicago, IL, USA).

## Results

### Demographic Characteristics in the study population

A total of 248 preterm infants with GA less than 32 weeks were enrolled in the study. 2 infants died before 36 weeks’ PMA and 3 infants had congenital heart disease. In the remaining 243 infants, 148(60.9%) were males and 95(39.1%) were females. The GA range from 25 to 31 weeks, with a mean GA of 29 weeks. Mean BW was 1418 ± 297 g. 79% of mothers were given prenatal steroids.

71 infants (29.2%) had BPD and 172 infants (70.8%) did not develop BPD during the study period. The clinical characteristics of infants with and without BPD are presented in Table 1. BPD patients had a lower GA and BW than non-BPD patients [28.9(25.6 − 31.7) weeks vs. 30.5(27.1 − 31.9) weeks, *p* < 0.001; 1185 ± 214g vs. 1515 ± 272g, *p* < 0.001; respectively]. Surfactant was used more often in BPD patients than non-BPD patients (95.8% vs. 54.1%, *p* < 0.001). The rate of early umbilical cord clamping (<30s) was higher in BPD patients than non-BPD patients (84.5% vs. 71.5%, p = 0.033). The incidence of PDA and ROP was significantly higher in BPD patients than non-BPD patients (83.1% vs. 66.9%, *p* = 0.011; 49.3% vs. 8.7%, *p* < 0.001; respectively). Rate of mechanical ventilation days more than 2 weeks was higher in BPD patients (94.4% vs. 13.4%, *p* < 0.001) and has significant difference. The number of transfusions in BPD patients was significantly greater than non-BPD patients [5(1 − 14) vs. 2(0 − 7), *p* < 0.001]. The days of hospital stay in BPD patients was longer than non-BPD patients (78 ± 21d vs. 46 ± 15d, *p* < 0.001). Gender, Apgar score at 1, 5, 10 min, rate of vaginal delivery, antibiotic use, prenatal steroids, maternal pregnancy-induced hypertension, maternal diabetes, premature rupture of membranes, and maternal age were similar between the two groups. No significant differences were found in incidence of pneumonia, NEC, IVH, and sepsis between the two groups.

### Hct between BPD-patients and non-BPD patients at different time points

**Figure 1:**
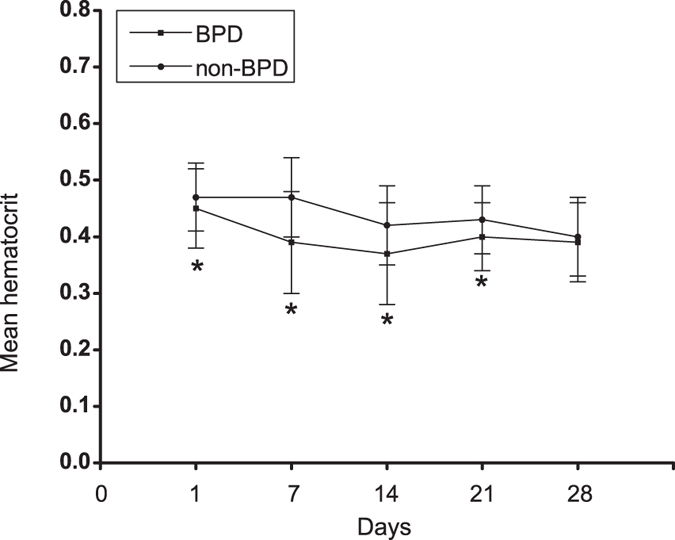
Comparision of hematocrit level between BPD patients and non-BPD patients at different time points. *p < 0.05.

There was a significant difference in Hct between BPD patients and non-BPD patients in 1d, 7d, 14d and 21d. Mean Hct was lower in BPD patients compared with non-BPD patients. However, there were no statistically significant differences in Hct between BPD patients and non-BPD patients in 28d. The mean Hct initially dropped, reaching a nadir at 14d, after which Hct slightly elevated in BPD patients and non-BPD patients (Fig. 1).

### Early anemia, late anemia and BPD

Out of the total 243 preterm infants, 165 (67.9%) were found to be anemic. Of 165 anemic patients, 70 (42.4%) had BPD, and out of 78 non-anemic patients, 1 (1.3%) had BPD, which was significantly different with a *p*-value of less than 0.001.

In the study, we chose 2 weeks as a cut-off point for the division of anemia into early anemia and late anemia. We found that rate of early anemic infants in BPD patients was higher than non-BPD patients (74.6% vs. 27.9%, *p* < 0.001). The incidence of late anemia was similar between BPD patients and non-BPD patients (Table 2).

To further evaluate the role of anemia on different grades of BPD, we compared the rates of anemia between severe BPD patients and non-severe BPD patients. As shown in Table 3, there is no difference in incidence of anemia, early anemia and late anemia between severe BPD and non-severe BPD patients.

The factors associated with the incidence of BPD in preterm infants in univariate analyses included GA, BW, surfactant use, PDA, days of mechanical ventilation >2 weeks, early umbilical cord clamping (<30 s), early anemia and number of transfusions. After controlling for significant risk factors, early anemia and number of transfusions were associated with increased risk of BPD rate using multivariate logistic regression. As shown in Table 4, the adjusted OR of early anemia for BPD was 4.891 (95% CI 1.568 − 15.257; *p* = 0.006), and the adjusted OR of number of transfusions for BPD was 1.703 (95% CI 1.249 − 2.322; *p* = 0.001).

## Discussion

Anemia is a common disease in preterm infants. Previous studies have reported significant association between anemia and respiratory disease. A prospective study conducted by Ramakishnan K. and his colleagues showed that anemia was a risk factor for childhood asthma^23^. Hussain SQ and coworkers reported that anemia was a risk factor for acute lower respiratory tract infections in children^24^. Until recently, there were no studies addressing anemia as a possible risk factor in BPD development. In the study, we firstly investigated the relationship between anemia and BPD using a case-control approach. We found that Hct was lower in infants at risk of BPD. As a variety of factors are associated with BPD, and many of the factors are related to anemia, separating the influences is difficult. However, after controlling the confounding variables, early anemia on the risk of developing BPD remained statistically significant.

In the study, we compared Hct levels between BPD patients and non-BPD patients at different time points. The results showed that mean Hct in BPD patients was lower than non-BPD patients in 1d, 7d, 14d and 21d. The results indicated that low Hct levels were closely related to BPD in preterm infants. The fact that Hct level in BPD patients dropped to a nadir at 14d may suggest the important role of Hct level in early life of preterm infants.

The underlying mechanism of the relationship between anemia and BPD may be explained as follows^15^,^16^,^17^,^18^. First, anemia may result in decreased oxygen carrying capacity of blood to lungs, resulting in increased anaerobic metabolism and high levels of lactic acid production. Second, anemia may decrease oxygen delivery to the respiratory center of the brain and cause respiratory symptoms, including tachypnea, dyspnea and apnea, which may increase days of supplemental oxygen or mechanical ventilation. Moreover, anemia may cause inflammation, which was also closely associated with the development of BPD.

We also found that early anemia was associated with the development of BPD. According to our study, the rate of early anemia in BPD patients was higher than non-BPD patients. The results also indicated that early anemia was an independent risk factor for BPD. After birth, oxygen dependency switches from placenta-based to lung-based and we speculate that anemia may impair the normal adaptation process, initiate a cascade of events leading to lung injury and finally result in the development of BPD. As time goes, older infants may tolerate anemia with no apparent clinical symptoms and reach a physiological balance. Therefore, early anemia may participate in the process of developing BPD. We compared the incidence of early anemia between severe BPD and non-severe BPD patients and found there were no significant differences. The finding implies that other factors other than early anemia may contribute to the progression of BPD.

Anemia is caused by many factors in preterm infants, including rapid postnatal growth, phlebotomy losses and delayed eternal feeding during hospitalization^15^,^16^. The optimal amount of protein supplementation for preterm infants can reduce incidence of anemia^15^,^16^. Delaying umbilical cord clamping has been shown to elevate birth Hemoglobin levels and decrease red blood cell (RBC) transfusion in preterm infants^25^,^26^. To reduce phlebotomy, it is necessary to decrease blood sampling volumes for laboratory tests, and avoid frequent blood sampling. Only those absolutely necessary laboratory testing should be performed. Non-invasive examination may also reduce blood loss. The implementation of restrictive transfusion guidelines is also necessary. Autologous transfusion with banked cord blood is a reasonable option, though most hospitals did not have the equipment to perform the therapy^27^. As plasma erythropietin (EPO) levels are relatively low in preterm infants, it is rational to consider rHuEPO as a potential treatment for anemia^28^,^29^,^30^. However, rHuEPO has not been widely used in NICU because of its uncertain efficacy^31^,^32^,^33^,^34^. Early use of supplemental of iron may probably reduce the incidence of anemia, however, the optimal dose and time of iron supplementation remains unclear and needs further research^35^.

Despite advances in determining the causes of anemia and in developing new treatment for anemia, RBC transfusion remains the primary treatment for anemia in preterm infants^36^. It is often given to maintain a certain Hct level believed to be desirable for infants’ clinical conditions^37^. However, we found that transfusion may be a risk factor for BPD in the study and it was consistent with previous reports^38^. Transfusion contains large amounts of iron, which increase iron levels in preterm infants. Elevated levels of free iron can produce oxygen free radicals, which may contribute to the development of BPD. In addition, transfusion impairs the normal erythropoietic response and reduces production of red cells^38^,^39^. Therefore, reducing the number of RBC transfusions in preterm infants may have significant beneficial effects. Guidelines for RBC transfusion varied widely from center to center and were mainly based on Hct or hemoglobin levels. Indications, time, and volume of blood transfusion remain controversial^40^,^41^,^42^. Therefore, it is important to determine an optimal level of Hct for transfusion and still requires further investigation.

Though ours is the first reported study to examine the influence of anemia in the development of BPD, there are some limitations. Although we controlled known confounding risk factors, there are other factors which may affect the development of BPD. In addition, more studies are needed to investigate the complicated mechanism of the effect of anemia on BPD development.

In conclusion, the study demonstrates an association between anemia and BPD in preterm infants. Early anemia (≤14days) is a significant risk factor for BPD. Medical treatments should be undertaken to prevent early anemia. A strict transfusion guideline is needed to reduce the incidence of BPD in our clinical practice.

## Additional Information

**How to cite this article**: Duan, J. *et al*. Association between anemia and bronchopulmonary dysplasia in preterm infants. *Sci. Rep.* **6**, 22717; doi: 10.1038/srep22717 (2016).

## Figures and Tables

**Table 1:** Comparison of clinical characteristics between BPD patients and non-BPD patients.

	**BPDpatients(n = 71)**	**non-BPDpatients(n = 172)**	***p*****value**
Gestational age(weeks)	28.9(25.6 − 31.7)	30.5(27.1 − 31.9)	<0.001
Birth weight (g)	1185 ± 214	1515 ± 272	<0.001
Male	50(70.4%)	98(57%)	0.051
Apgar score at 1 min	8(2 − 10)	8(2 − 10)	0.544
Apgar score at 5 min	9(2 − 10)	9(3 − 10)	0.219
Apgar score at 10 min	9(2 − 10)	9(3 − 10)	0.113
Vaginal delivery	39(54.9%)	88(51.2%)	0.593
Prenatal steroids	53(74.6%)	139(80.8%)	0.283
Maternal pregnancy-induced hypertension	16(22.5%)	32(18.6%)	0.484
Maternal diabetes	16(22.5%)	34(19.8%)	0.627
Premature rupture of membranes	23(32.4%)	77(44.8%)	0.075
Maternal age	29 ± 4	30 ± 5	0.430
Antibiotic use	17(23.9%)	54(31.4%)	0.245
Early umbilical cord clamping (<30s)	60(84.5%)	123(71.5%)	0.033
Surfactant use	68(95.8%)	93(54.1%)	<0.001
Days of mechanical ventilation >2 weeks	67(94.4%)	23(13.4%)	<0.001
Pneumonia	50(70.4%)	106(61.6%)	0.193
Sepsis	10(14.1%)	12(7.0%)	0.079
Patent ductus arteriosus	59(83.1%)	115(66.9%)	0.011
Necrotizing enterocolitis	4(5.6%)	9(5.2%)	1.000
Intaventricular hemorrhage	27(38%)	46(26.7%)	0.081
Retinopathy of prematurity	35(49.3%)	15(8.7%)	<0.001
Number of transfusions	5(1 − 14)	2(0 − 7)	<0.001
Length of hospital stay (days)	78 ± 21	46 ± 15	<0.001

**Table 2:** Rate of anemia between BPD patients and non-BPD patients.

	**BPD patients (n = 71)**	**Non-BPD patients (n = 172)**	***p*****value**
Anemia	70(98.6%)	95(55.2%)	<0.001
Early anemia	53(74.6%)	48(27.9%)	<0.001
Late anemia	17(23.9%)	47(27.3%)	0.586

**Table 3:** Rate of anemia between severe BPD patients and non-severe BPD patients.

	**Severe BPDpatients (n = 16)**	**Non-severe BPDpatients (n = 55)**	***p*****value**
Anemia	16(100%)	54(98.2%)	1.000
Early anemia	13(81.3%)	40(72.7%)	0.716
Late anemia	3(18.8%)	14(25.5%)	0.826

**Table 4:** Risk factors for BPD by multiple logistic regression analysis.

	**Adjust odds ratio**	**95% Confidence interval**	***p*** **value**
Gestational age	0.774	0.458 − 1.306	0.336
Birth weight	0.999	0.996 − 1.002	0.385
Surfactant use	1.915	0.261 − 14.074	0.523
Patent ductus arteriosus	0.630	0.164 − 2.415	0.500
Days of mechanical ventilation >2 weeks	29.978	7.474 − 120.239	<0.001
Early umbilical cord clamping (<30s)	2.030	0.460 − 8.952	0.350
Early anemia	4.891	1.568 − 15.257	0.006
Number of transfusions	1.703	1.249 − 2.322	0.001

## References

[b1] Truog W. E. Bronchopulmonary dysplasia: another step along the path. J. Pediatr. 166, 521–522 (2015). 25556019 10.1016/j.jpeds.2014.11.028

[b2] Strueby L. & Thebaud B. Advances in bronchopulmonary dysplasia. Expert Rev. Respir. Med. 8, 327–338 (2014). 24666156 10.1586/17476348.2014.899907

[b3] Zysman-Colman Z., Tremblay G. M., Bandeali S. & Landry J. S. Bronchopulmonary dysplasia - trends over three decades. Paediatr. Child Health 18, 86–90 (2013). 24421662 10.1093/pch/18.2.86 PMC3567902

[b4] Popova A. P. Mechanisms of bronchopulmonary dysplasia. J. Cell Commun. Signal. 7, 119–127 (2013). 23334556 10.1007/s12079-013-0190-x PMC3660689

[b5] Hayes D. Jr. Feola D. J., Murphy B. S., Shook L. A. & Ballard H. O. Pathogenesis of bronchopulmonary dysplasia. Respiration 79, 425–436 (2010). 19786727 10.1159/000242497

[b6] Poindexter B. B. & Jobe A. H. The Diagnostic Conundrum of Bronchopulmonary Dysplasia. J. Pediatr. 167, 517–518 (2015). 26138878 10.1016/j.jpeds.2015.06.029

[b7] Niedermaier S. & Hilgendorff A. Bronchopulmonary dysplasia - an overview about pathophysiologic concepts. Mol. Cell Pediatr . 2, 2 (2015). 26542292 10.1186/s40348-015-0013-7 PMC4530566

[b8] Martin R. J., Di Fiore J. M. & Walsh M. C. Hypoxic Episodes in Bronchopulmonary Dysplasia. Clin. Perinatol. 42, 825–838 (2015). 26593081 10.1016/j.clp.2015.08.009 PMC4660265

[b9] Keszler M. & Sant’Anna G. Mechanical Ventilation and Bronchopulmonary Dysplasia. Clin. Perinatol. 42, 781–796 (2015). 26593078 10.1016/j.clp.2015.08.006

[b10] Somaschini M. . Genetic predisposing factors to bronchopulmonary dysplasia: preliminary data from a multicentre study. J. Matern. Fetal Neonatal Med. 25 Suppl 4, 127–130 (2012). 22958043 10.3109/14767058.2012.714995

[b11] Schena F. . Association between Hemodynamically Significant Patent Ductus Arteriosus and Bronchopulmonary Dysplasia. J. Pediatr. 166, 1488–1492 (2015). 25882876 10.1016/j.jpeds.2015.03.012

[b12] Demirel N., Bas A. Y. & Zenciroglu A. Bronchopulmonary dysplasia in very low birth weight infants. Indian J. Pediatr. 76, 695–698 (2009). 19381510 10.1007/s12098-009-0110-5

[b13] Ali Z., Schmidt P., Dodd J. & Jeppesen D. L. Bronchopulmonary dysplasia: a review. Arch. Gynecol. Obstet. 288, 325–333 (2013). 23420126 10.1007/s00404-013-2753-8

[b14] Ozsoylu S. Iron deficiency anemia in late preterm infants. Turk. J. Pediatr. 56, 119 (2014). 24946367

[b15] Ozdemir H. . Iron deficiency anemia in late-preterm infants. Turk. J. Pediatr. 55, 500–505 (2013). 24382530

[b16] Von Kohorn I. & Ehrenkranz R. A. Anemia in the preterm infant: erythropoietin versus erythrocyte transfusion–it’s not that simple. Clin. Perinatol. 36, 111–123 (2009). 19161869 10.1016/j.clp.2008.09.009 PMC2683173

[b17] Elguazzar S., Alaoui A. M. & Izgua A. T. [Evaluation of the practice of transfusion in the anemia in preterm infants]. Rev. Med. Brux. 34, 4–11 (2013). 23534309

[b18] Sallmon H. & Sola-Visner M. Clinical and research issues in neonatal anemia and thrombocytopenia. Curr. Opin. Pediatr. 24, 16–22 (2012). 22227780 10.1097/MOP.0b013e32834ee5cc

[b19] Walsh M. C. & Kliegman R. M. Necrotizing enterocolitis: treatment based on staging criteria. Pediatr. Clin. North Am. 33, 179–201 (1986). 3081865 10.1016/S0031-3955(16)34975-6 PMC7131118

[b20] Jordan C. O. Retinopathy of prematurity. Pediatr. Clin. North Am. 61, 567–577 (2014). 24852153 10.1016/j.pcl.2014.03.003

[b21] Singh R. . Association of necrotizing enterocolitis with anemia and packed red blood cell transfusions in preterm infants. J. Perinatol. 31, 176–182 (2011). 21273983 10.1038/jp.2010.145 PMC3234132

[b22] Jobe A. H. & Bancalari E. Bronchopulmonary dysplasia. Am. J. Respir. Crit. Care Med. 163, 1723–1729 (2001). 11401896 10.1164/ajrccm.163.7.2011060

[b23] Ramakrishnan K. & Borade A. Anemia as a risk factor for childhood asthma. Lung India 27, 51–53 (2010). 20616934 10.4103/0970-2113.63605 PMC2893424

[b24] Hussain S. Q., Ashraf M., Wani J. G. & Ahmed J. Low Hemoglobin Level a Risk Factor for Acute Lower Respiratory Tract Infections (ALRTI) in Children. J. Clin. Diagn. Res. 8, PC01–03 (2014). 24959486 10.7860/JCDR/2014/8387.4268 PMC4064840

[b25] Chiruvolu A. . Effect of delayed cord clamping on very preterm infants. Am. J. Obstet. Gynecol. 213, 676 e671–677 (2015). 10.1016/j.ajog.2015.07.016 26196456

[b26] Strauss R. G. . A randomized clinical trial comparing immediate versus delayed clamping of the umbilical cord in preterm infants: short-term clinical and laboratory endpoints. Transfusion 48, 658–665 (2008). 18194383 10.1111/j.1537-2995.2007.01589.x PMC2883857

[b27] Eichler H. . Cord blood as a source of autologous RBCs for transfusion to preterm infants. Transfusion 40, 1111–1117 (2000). 10988315 10.1046/j.1537-2995.2000.40091111.x

[b28] Ohls R. K., Li Y., Trautman M. S. & Christensen R. D. Erythropoietin production by macrophages from preterm infants: implications regarding the cause of the anemia of prematurity. Pediatr. Res. 35, 169–170 (1994). 8165050 10.1203/00006450-199402000-00008

[b29] Saleh M. I., Nalbant D., Widness J. A. & Veng-Pedersen P. Population pharmacodynamic analysis of erythropoiesis in preterm infants for determining the anemia treatment potential of erythropoietin. Am. J. Physiol. Regul. Integr. Comp. Physiol . 304, R772–781 (2013). 23485870 10.1152/ajpregu.00173.2012 PMC3652082

[b30] Zuppa A. A. . Comparison between two treatment protocols with recombinant human erythropoietin (rHuEpo) in the treatment of late anemia in neonates with Rh-isoimmunization. Pediatr. Med. Chir. 34, 186–191 (2012). 23173411 10.4081/pmc.2012.72

[b31] Yasmeen B. H. . Effect of short-term recombinant human erythropoietin therapy in the prevention of anemia of prematurity in very low birth weight neonates. Bangladesh Med. Res. Counc. Bull. 38, 119–123 (2012). 23540189 10.3329/bmrcb.v38i3.14340

[b32] Rosebraugh M. R., Widness J. A., Nalbant D., Cress G. & Veng-Pedersen P. Pharmacodynamically optimized erythropoietin treatment combined with phlebotomy reduction predicted to eliminate blood transfusions in selected preterm infants. Pediatr. Res. 75, 336–342 (2014). 24216541 10.1038/pr.2013.213 PMC4418561

[b33] Ohlsson A. & Aher S. M. Early erythropoietin for preventing red blood cell transfusion in preterm and/or low birth weight infants. Cochrane Database Syst. Rev. 4, CD004863 (2014). 24771408 10.1002/14651858.CD004863.pub4

[b34] Aher S. M. & Ohlsson A. Late erythropoietin for preventing red blood cell transfusion in preterm and/or low birth weight infants. Cochrane Database Syst. Rev. 4, CD004868 (2014). 24760628 10.1002/14651858.CD004868.pub4

[b35] Mills R. J. & Davies M. W. Enteral iron supplementation in preterm and low birth weight infants. Cochrane Database Syst. Rev. 3, CD005095 (2012). 22419305 10.1002/14651858.CD005095.pub2 PMC11528245

[b36] dos Santos A. M. . Factors associated with red blood cell transfusions in very-low-birth-weight preterm infants in Brazilian neonatal units. BMC Pediatr. 15, 113 (2015). 26341125 10.1186/s12887-015-0432-6 PMC4560891

[b37] Bell E. F. Transfusion thresholds for preterm infants: how low should we go? J. Pediatr. 149, 287–289 (2006). 16939732 10.1016/j.jpeds.2006.06.033

[b38] Zhang Z., Huang X. & Lu H. Association between red blood cell transfusion and bronchopulmonary dysplasia in preterm infants. Sci. Rep. 4, 4340 (2014). 24614152 10.1038/srep04340 PMC3949297

[b39] Bak S. Y., Lee S., Park J. H., Park K. H. & Jeon J. H. Analysis of the association between necrotizing enterocolitis and transfusion of red blood cell in very low birth weight preterm infants. Korean J. Pediatr. 56, 112–115 (2013). 23559972 10.3345/kjp.2013.56.3.112 PMC3611044

[b40] McCoy T. E. . Neurocognitive profiles of preterm infants randomly assigned to lower or higher hematocrit thresholds for transfusion. Child Neuropsychol. 17, 347–367 (2011). 21360360 10.1080/09297049.2010.544647 PMC3115491

[b41] Chirico G., Beccagutti F., Sorlini A., Motta M. & Perrone B. Red blood cell transfusion in preterm infants: restrictive versus liberal policy. J. Matern. Fetal Neonatal. Med. 24 Suppl 1, 20–22 (2011). 21942584 10.3109/14767058.2011.607566

[b42] Gueguen J., Beuchee A., Gaillot T., Betremieux P. & Pladys P. Red cell transport and transfusion in preterm infants. Arch. Dis. Child. Fetal Neonatal. Ed . 94, F229 (2009). 19383860 10.1136/adc.2008.146621

